# Hypertensive Heart Disease—The Imaging Perspective

**DOI:** 10.3390/jcm12093122

**Published:** 2023-04-25

**Authors:** Tevfik F. Ismail, Simon Frey, Beat A. Kaufmann, David J. Winkel, Daniel T. Boll, Michael J. Zellweger, Philip Haaf

**Affiliations:** 1 King’s College London & Cardiology Department, School of Biomedical Engineering and Imaging Sciences, Guy’s and St Thomas’ NHS Foundation Trust, London SE1 7EH, UK; 2 Department of Cardiology and Cardiovascular Research Institute Basel (CRIB), University Hospital Basel, University of Basel, Petersgraben 4, CH-4031 Basel, Switzerland; 3 Department of Radiology, University Hospital Basel, University of Basel, CH-4031 Basel, Switzerland

**Keywords:** hypertensive heart disease, echocardiography, cardiovascular magnetic resonance, computed tomography, myocardial perfusion scintigraphy, PET, hypertrophic cardiomyopathy, cardiac amyloidosis, Fabry’s disease

## Abstract

Hypertensive heart disease (HHD) develops in response to the chronic exposure of the left ventricle and left atrium to elevated systemic blood pressure. Left ventricular structural changes include hypertrophy and interstitial fibrosis that in turn lead to functional changes including diastolic dysfunction and impaired left atrial and LV mechanical function. Ultimately, these changes can lead to heart failure with a preserved (HFpEF) or reduced (HFrEF) ejection fraction. This review will outline the clinical evaluation of a patient with hypertension and/or suspected HHD, with a particular emphasis on the role and recent advances of multimodality imaging in both diagnosis and differential diagnosis.

## 1. Introduction

Worldwide, the age-standardised prevalence of hypertension continues to rise and has reached 30% in the general population and up to two-thirds in those over the age of 60 [1,2]. Hypertension is the most frequent cardiovascular risk factor that is associated with cardiac remodelling [1,2]. Hypertensive heart disease (HHD) is the spectrum of cardiovascular abnormalities developing in response to the chronic exposure of the left ventricle (LV) and left atrium (LA) to elevated systemic blood pressure. Hypertension is an important vascular risk factor for the development of atherosclerosis and its sequelae.

Left ventricular hypertrophy (LVH)—defined as an increase in the LV wall thickness or mass—is widely regarded as a cardinal feature and phase in the evolution of HHD [3]. Traditional models of the pathophysiological response to chronic hypertension describe the development of LVH as an initial adaptive response of the LV to reduce wall stress. According to these models, systolic heart failure then develops via the intermediate step of LVH when the ability of the ventricle to compensate for elevated wall stress is exhausted or the ventricle is exposed to another additional insult [3]. However, while this transition can doubtless occur in some individuals, it is increasingly appreciated that these models are not supported by empirical evidence in humans and that concentric LVH does not commonly transition into dilated systolic heart failure in the absence of another myocardial insult (e.g., intercurrent myocardial infarction) [3]. Some patients with hypertension also appear to be able to develop dilated systolic heart failure in the absence of antecedent concentric LVH or an additional intercurrent myocardial insult, and indeed, eccentric remodelling rather than concentric hypertrophy may occur in patients with hypertension [4].

LV structural changes include hypertrophy and interstitial fibrosis that in turn lead to functional changes including diastolic dysfunction and impaired LA and LV mechanical function. Ultimately, these changes can lead to heart failure with a preserved (HFpEF) or reduced (HFrEF) ejection fraction [5]. It should be noted that while hypertrophy is a hallmark of HHD, interstitial fibrosis and its functional consequences may precede the development of hypertrophy. Synergistically, diabetes, obesity, dyslipidaemia, metabolic syndrome, obstructive sleep apnoea, and renal failure add to more advanced LV dysfunction and structural impairment [6]. A further challenge to clinicians is the differential diagnosis of LVH, particularly where dual pathology may exist. This review will outline the clinical evaluation of a patient with hypertension and/or suspected hypertensive heart disease, with a particular emphasis on the role of multimodality imaging in both diagnosis and differential diagnosis.

## 2. History and Examination

Most patients with hypertension are asymptomatic and, as such, the condition has often been described as a silent killer [7]. History taking should therefore focus on symptoms that may result from a hypertensive crisis (headache, dizziness, visual disturbance, chest pain, dyspnoea), conditions which may predispose to or contribute to hypertension (e.g., phaeochromocytoma, Cushing’s disease/syndrome, acromegaly, coarctation of the aorta, etc.) and detecting hypertensive heart failure (exertional dyspnoea, orthopnoea, paroxysmal nocturnal dyspnoea, oedema, paroxysmal palpitations/atrial fibrillation). Patients should be comprehensively examined to diagnose hypertension, identify syndromic causes (Table 1) or risk factors for secondary hypertension, and screen for cardiovascular sequelae [8]. Table 2 summarises the key clinical features to assess.

## 3. Investigations

All patients should have a baseline 12-lead Electrocardiogram to screen for LVH/end-organ damage although it has a low sensitivity for detecting LVH [8]. All patients should also be offered basic blood chemistry to screen for renal disease and markers that might point towards a secondary cause (e.g., unexplained hypokalaemia). Most patients over the age of 40 who have confirmed hypertension and who respond to treatment do not need any further investigations. Patients under the age of 40 without a clear cause for their hypertension should be screened for secondary causes. Those over the age of 40 who have suggestive features on history taking, examination, or initial biochemical screening should also be considered for further investigation. Testing for adrenal secondary causes for hypertension should focus on a biochemical confirmation of diagnosis first before any imaging is undertaken given the high incidence of often unrelated adrenal adenomas in the general population. Non-invasive imaging of the renal arteries with MRI or CT should be considered to screen for fibromuscular dysplasia, and in older patients who experience significant declines in renal function on ACE-inhibitor/angiotensin II receptor blocker therapy, to screen for atherosclerotic renal artery stenosis. A urine sample should be obtained to screen for microalbuminuria/proteinuria and glycosuria. A 24 h collection can be of value to assess sodium excretion (and thereby identify dietary sodium excess) or separately for catecholamines where a phaeochromocytoma is suspected.

## 4. Echocardiography

Given its availability and low cost, transthoracic echocardiography (TTE) is the first-line diagnostic imaging test to detect structural and functional changes in HHD. Left ventricular mass index (LVMI), left ventricular diastolic dysfunction (LVDD), and impaired global longitudinal strain (GLS) have prognostic implications in patients with arterial hypertension [9,10,11]. However, since large outcome trials show lower cardiovascular event rates that parallel pharmacologic blood pressure lowering, antihypertensive therapy is warranted irrespective of the presence of HHD and so routine echocardiography in all patients is not indicated.

**Figure 1:**
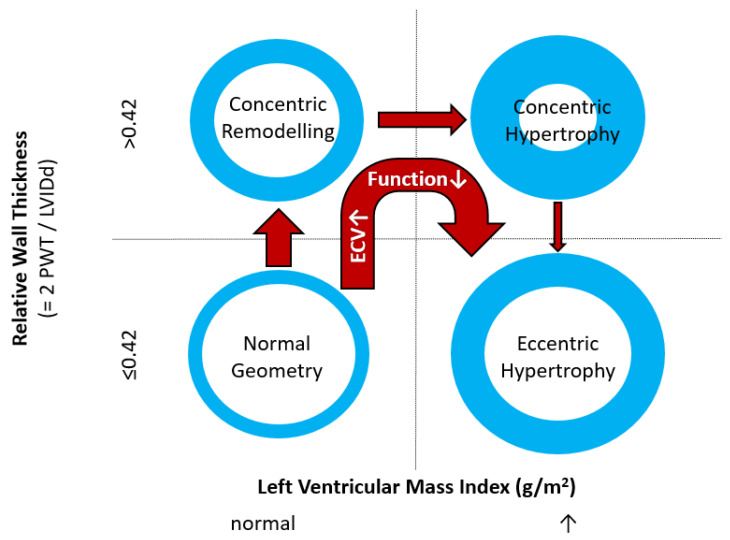
Commonly used classification of left ventricular geometry and typical geometry changes occurring in patients with uncontrolled hypertension/hypertensive heart disease. PWT denotes posterior wall thickness; LVIDd denotes Left Ventricular Inner Diastolic diameter; ECV denotes myocardial extracellular volume. Deteriorating function implies both parameters of systolic and diastolic function. Normal values for Left Ventricular Mass Index depend on gender and imaging modality used.

Echocardiographic assessment of LVH and thus left ventricular mass (LVM) relies on linear measurements of LV dimensions on 2-dimensional images. Calculation of myocardial mass then uses geometric assumptions with respect to LV shape [12], which, together with the dependence on image quality, represents a major drawback of this technique. Thus, while echocardiographic assessment of LVM in populations can detect, for example, differences in subpopulations, its sensitivity to detect changes on an individual level is reduced. When compared to cardiovascular magnetic resonance (CMR) imaging, 2-dimensional echocardiography tends to overestimate LVM in patients with good image quality and underestimate LVM in those with limited image quality [13]. The aforementioned limitations regarding geometric assumptions can be overcome by using 3-dimensional echocardiography, and it has been shown that this leads to LVH measurements that reduce underestimation compared to CMR [14]. However, 3D echocardiography heavily depends on optimal image quality. Since LVM is related to body size, indexing values to parameters of body size such as height or body surface area is necessary for comparison to population-based reference values. Whereas LVM/body surface area ratios underestimate the prevalence of LVH in obese patients, LVM/height ratios may need to rely on additional allometric scaling for accuracy [15]. Depending on the relation between LVM and LV dimensions, LVH can be further divided into concentric and eccentric forms (Figure 1). Together with LV remodelling without LVH, which represents an initial stage of HHD, these parameters are associated with increased cardiovascular risk.

LV systolic function is maintained at normal or near-normal values until the late stages of HHD. The most commonly used surrogate marker of LV systolic function is left ventricular ejection fraction (LVEF), which depends not only on LV contractility but also on pre- and afterload and left atrial function. In clinical practice, LVEF is measured on 2D echocardiography using Simpson’s biplane method or on 3D echocardiography using manually assisted semi-automated endocardial border tracking [12]. Of note, fully automated artificial-intelligence-supported software tools have recently been shown to accurately reproduce LVEF measurements performed by human experts [16].

While LVEF may remain normal along the natural history of HHD, echocardiographic techniques such as speckle tracking strain have been shown to be able to detect subtler impairments in myocardial mechanics such as reduced global longitudinal strain (GLS) [17]. In hypertensive patients with normal ejection fraction, reduced GLS has been shown to predict cardiovascular events independent of clinical parameters and the presence of increased LVM [11]. The myocardial consequences of chronic systolic and diastolic hypertension are not confined to myocyte hypertrophy but also include perivascular and intramyocardial diffuse fibrosis. The LV strain has emerged as a sensitive marker of LV function and may provide a long-term prognostic assessment over traditional echocardiographic parameters [18]. In patients with HHD, the LV strain (specifically GLS) may predict MACE (death and admission because of heart failure, myocardial infarction, and strokes), independent of and incremental to clinical parameters and concentric hypertrophy [11]. While GLS represents the best validated and most easily measured parameter of LV mechanics, multidirectional strain and LV twist/untwist are also affected during the pathogenesis of HHD with an early increase in twist followed by deterioration in later stages [19].

Inherent to the LV structural changes including hypertrophy and interstitial fibrosis, LV elasticity, compliance, and stiffness are compromised in hypertensive patients and lead to LVDD. While a complete description of an echocardiographic assessment of diastolic function is beyond the scope of the present review, measurements include Doppler and tissue Doppler measurements of mitral inflow and mitral annular velocities [20]. These measurements are then integrated with LA volumes and systolic pulmonary pressure to diagnose LVDD. However, the accuracy of these parameters is controversial, with some reports showing a good correlation with invasive pressure measurements [21], while others have reported only modest relationships [22].

The LA dilates as a consequence of LVDD in HHD, and the LA volume index (LAVI) has been shown to independently predict cardiovascular events [23]. Recently, speckle tracking strain imaging has enabled accurate assessment of LA mechanics throughout the cardiac cycle. This includes LA reservoir function during LV contraction and isovolumic relaxation, conduit function during early and mid-diastole, and active atrial contraction following atrial systole. In hypertensive patients, limited data indicate that reservoir and conduit function decreases, while, likely as a compensatory mechanism to maintain stroke volume, active atrial contraction increases [24].

Furthermore, arterial hypertension is a risk factor for developing an abdominal aortic aneurysm. Screening for abdominal aortic aneurysms can easily be integrated into a routine echocardiography assessment and is indicated in patients with long-standing hypertension and individuals over 65 years of age (female smokers and males irrespective of smoking status) [25].

Serial echocardiography can be used in principle to monitor longitudinal changes in LV mass in response to treatment, which may have prognostic implications; however, the higher accuracy and precision of CMR make this more suited for this application.

## 5. Cardiovascular Magnetic Resonance

Hypertensive patients with LV hypertrophy disproportionate to the degree of hypertension may be referred for CMR for further examination and differential diagnosis. CMR is regarded as the non-invasive gold standard for the evaluation of LV volumes, systolic function, LV mass, and myocardial tissue characterisation [26]. Image acquisition can be achieved largely independent of body habitus, imaging windows, and without ionising radiation exposure [26]. Cardiac images are usually acquired in four-, three-, and two-chamber views, as well as a full stack of 10–15 short-axis images. Its three-dimensional nature with excellent spatial resolution and high tissue contrast enables accurate measurement of cardiac function and morphology: LV volumes, mass, and the ejection fraction, as well as an assessment of regional wall motion abnormalities without relying on geometrical assumptions (such as Simpson’s biplane method) [27]. CMR provides the potential to also assess and quantify focal and diffuse myocardial fibrosis caused by hypertension (hypertension-mediated organ damage) and to screen for secondary causes of hypertension [28]. The nascent technique of cardiac MR elastography can also allow the non-invasive measurement of myocardial stiffness [29]. Importantly, the high accuracy and precision of CMR allow prognostically important longitudinal changes in LV mass in response to therapy to be readily appreciated [30]

Table 3 summarises a suggested CMR scan protocol for the diagnosis of presumed HHD and differential diagnosis of patients with a “thickened left ventricle”.

**Figure 2:**
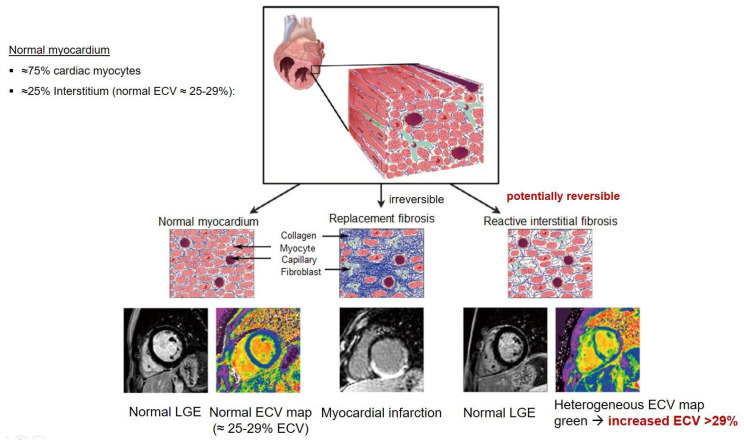
Tissue characterization with Late Gadolinium Enhancement (LGE) and Extracellular Volume (ECV) Map. Adapted with permission from [33].

Similar to echocardiography, LV geometry can be assessed using relative wall thickness (RWT) and CMR-specific normal values for LV myocardial mass (Figure 2). LA dimensions can be assessed with Simpson’s biplane or area-length methods from the standard views, or more precisely with a volumetric assessment using an atrial short-axis stack. LA enlargement is a reliable marker of diastolic dysfunction (chronically elevated LV filling pressures) in the absence of mitral valve disease [31]. The recently introduced left atrial coupling index (LACI) is a ratio of the indexed left atrial end-diastolic volume (LAVI) in relation to the left ventricular end-diastolic volume (LVEDVI) [32]. This ratio may offer prognostic information regarding cardiovascular events such as atrial fibrillation, heart failure, and coronary artery disease-related death. 

## 6. CMR Strain

Feature-tracking strain imaging can also be performed in CMR with the advantage of better image quality, lack of angle dependency or impediment from difficult acoustic windows, and less observer dependency [34]. Myocardial strain exhibited good reproducibility, declined prior to changes in LVEF or volumes, and correlated with mean arterial pressure in hypertensive patients [34]. LV longitudinal strain exhibits better reproducibility and less vendor dependency compared to the other strain types [6]. Furthermore, the CMR strain was shown to correlate with myocardial fibrosis detected by CMR [35].

## 7. CMR Stress Perfusion

Hypertension is a well-established atherosclerotic risk factor for the evolution of coronary artery disease (CAD). Both macrovascular CAD of the epicardial coronary arteries and microvascular CAD (small vessel disease with endothelial dysfunction) are detectable by CMR stress perfusion [36]. There is an increased frequency of hypertension in patients with chest pain, angiographically normal coronary arteries, and microvascular CAD, which may be detected by CMR using visual assessment or perfusion quantification techniques [31].

CMR stress perfusion essentially visualizes myocardial first-pass perfusion during pharmacological stress. Images are interpreted in conjunction with rest perfusion images and late gadolinium enhancement (LGE) images. In the presence of a significant epicardial coronary stenosis, the myocardial contrast uptake is reduced in a specific coronary territory (coronary pattern). Conversely, microvascular dysfunction displays a more diffuse and delayed but synchronous myocardial contrast uptake during stress. Stress CMR offers excellent sensitivity and specificity for the detection of anatomically and functionally significant CAD [37] and allows risk stratification irrespective of LVEF, the presence of CAD, symptoms, and LGE [38]. The assessment of quantitative myocardial blood flow has prognostic value and is likely to be used in the clinical routine in the near future [39]. 

## 8. Tissue Characterisation with CMR

To avoid the low but important risks of endomyocardial biopsy, which can have an overall complication rate of up to 6% [2], myocardial fibrosis can be assessed non-invasively using CMR: LGE is suitable for detecting irreversible replacement fibrosis and myocardial scarring and T1/ECV mapping for detection of potentially reversible (reactive) interstitial and more diffuse fibrosis (not detectable by LGE) (Figure 2).

## 9. Tissue Characterisation with Late Gadolinium Enhancement

LGE has become the reference standard for non-invasive imaging of myocardial scar and focal fibrosis [40]. Gadolinium chelates are interstitial agents that cannot penetrate healthy intact cell membranes. Therefore, they remain in the interstitial space and accumulate in areas of cell injury/necrosis and focal fibrosis where this is expanded, while in healthy regions, contrast more readily washes out [41]. Specific LGE patterns are seen in different diseases (e.g., subendocardial fibrosis in CAD, patchy epicardial/mid-wall fibrosis in areas of hypertrophy in HCM). Minor areas of LGE can be detected in up to 50% of patients with HHD, but there is no specific pattern (in 95% of the non-ischaemic LGE distribution) [42]. If present, LGE is often found in the basal to mid-septal, inferior, and inferolateral segments in patients with HHD [42]. The severity of diastolic dysfunction increases with the extent of fibrosis by LGE [25,31]. Furthermore, focal fibrosis/LGE may be a substrate for ventricular arrhythmia and is associated with sudden cardiac death [31].

## 10. Tissue Characterisation with T1 and Extracellular Volume (ECV) Mapping

LGE allows the detection of focal alterations in the myocardium, but diffuse fibrosis may go undetected on LGE imaging. Tissue characterisation with parametric mapping methods such as T1 and ECV mapping has the potential to detect and quantify both focal and diffuse alterations in the myocardial structure. Furthermore, changes in the myocardium over time may be assessed longitudinally [40]. Estimation of myocardial ECV (interstitium and extracellular matrix) requires the measurement of myocardial and blood *T*1 before and after the administration of contrast agents, as well as the patient’s haematocrit. *ECV* can then be calculated using the formula:

$$ECV=\left(1-haematocrit\right)\frac{\frac{1}{post\;contrast\;T1\;myo}-\frac{1}{native\;T1\;myo}}{\frac{1}{post\;contrast\;T1\;blood}-\frac{1}{native\;T1\;blood}}$$

Myocytes account for approximately one-third of all cells in normal myocardium. The remaining two-thirds of cells include endothelial and vascular smooth muscle cells and fibroblasts in interstitial/perivascular spaces [2] (Figure 2). Normal CMR *ECV* values vary between 25.3 and 3.5% [43]. Ideally, age- and sex-corrected normal values for *ECV* should be used [44]. Hypertension affects both the cellular and extracellular compartments of the myocardium. In addition to cardiomyocyte hypertrophy, in HHD, fibrous tissue (primarily type I fibrillar collagen) is deposited in the extracellular matrix over time and leads to increased tissue stiffness (i.e., diastolic dysfunction) [2]. *ECV* values are higher in hypertensive patients with LVH than in patients without LVH, and eccentric forms of hypertrophy seem to have the most fibrosis and highest *ECV* values, together with more pronounced systolic impairment and are associated with a poor cardiovascular prognosis (Figure 1) [2,6]. CMR-derived *T*1 mapping and strain analysis seem to be related, but an adequate comparison of the performance of these parameters is often limited due to the lack of harmonization of measurement methods [35]. Furthermore, *ECV* values seem to correlate with many blood biomarkers associated with (i) systemic inflammation; (ii) metabolism; (iii) fibrosis; (iv) chemotaxis; and (v) platelet activation [6]. This may suggest that an increase in *ECV* in hypertensive patients is a (non-specific) imaging biomarker of inflammation, tissue remodelling, atherogenesis, or metabolic disorder in patients with HHD [6]. Given the clinical consequences of myocardial fibrosis in HHD and considering the potential for recovery of fibrosis with appropriate treatment, the need for an accurate diagnosis of myocardial fibrosis is apparent.

Although tissue characterisation with native *T*1 and *ECV* has been shown to have incremental diagnostic benefits even in very early disease stages (e.g., diffuse fibrosis not detectable by LGE), there is an overlap between different cardiomyopathies and some overlap with normal T1 values. The difference in ECV between normal subjects and patients with HHD is small (0.29 ± 0.03 vs. 0.27 ± 0.02) [2]. Furthermore, other pathologies in addition to fibrosis increase ECV values such as myocardial inflammation or amyloid deposition. As with all medical parameters, abnormalities in native *T*1 and *ECV* need to be interpreted within their clinical context and pre-test probabilities and in conjunction with established CMR techniques such as LGE. Nevertheless, native *T*1 and myocardial *ECV* mapping seem to be promising imaging biomarkers to characterise HHD and eventually may even help to guide and monitor treatment response with antifibrotic agents in certain hypertensive individuals. Non-ischaemic LGE is associated with adverse LV remodelling, worse function, and elevated markers of wall stress and myocardial injury. Reactive interstitial fibrosis is potentially reversible with targeted therapies [45]. It is increasingly appreciated that the identification of focal or diffuse fibrosis may have significant independent prognostic implications and may help in monitoring disease progression and guiding anti-fibrotic therapies in the future [46]. 

## 11. Phenocopies and Differential Diagnosis of Hypertensive Heart Disease

The differential diagnosis of HHD includes hypertrophic cardiomyopathy and its phenocopies (e.g., Fabry’s disease, mitochondrial disease); valvular heart disease (sub-valvular, valvular, and supra-valvular aortic stenosis); pseudohypertrophy (amyloidosis (Table 4A); sarcoidosis (Table 4B)); and the so-called athletic heart, among others. Imaging can plan an important role in elucidating the cause of LVH [47], especially in patients with dual-presence arterial hypertension/HHD and another cardiomyopathy associated with LV hypertrophy.

Echocardiography allows the detailed evaluation of the left ventricular outflow tract, aortic valve, and supra-valvular structures. The combination with Doppler imaging allows haemodynamically significant obstructions to be readily detected and quantified. Valvular contributions to LVH can therefore be readily identified. However, CMR or DPD-scintigraphy can offer added value as ATTR amyloidosis and aortic stenosis frequently coexist [48].

Cardiovascular magnetic resonance (CMR) uniquely allows tissue characterisation to be undertaken and as such, can readily differentiate hypertensive LVH from causes of pseudohypertrophy or thickening of the left ventricle due to myocardial infiltration with amyloid (Table 4A) or sarcoid (Table 4B) [47].

All imaging findings should be assessed in their clinical context; patient history, cardiac blood biomarkers, and a 12-lead ECG can be very helpful in the differential diagnosis of LV hypertrophy (Table 5 and Supplement Table S1).

## 12. Cardiac Amyloid

Cardiac amyloid is typically characterised by the thickening of the ventricular walls, atria, and valvular structures (Table 4A). There is usually reduced long-axis motion. Native T1 and extracellular volume (ECV) are increased, and the latter may potentially be used as a marker of the degree of infiltration, and to monitor response to therapy. The findings on late gadolinium enhancement imaging are characteristic of abnormal gadolinium kinetics (the myocardium nulling in advance of the blood pool), difficulty in achieving a suitable null time, and widespread circumferential subendocardial enhancement [49]. When the latter involves the LV sub-endocardium and the RV aspect of the septum, it can give rise to the so-called zebra-stripe sign (Table 4C) [49]. The LV thickening seen in amyloid is often described as concentric, but asymmetrical patterns of thickening not too dissimilar to the patterns seen in hypertrophic cardiomyopathy are not infrequently encountered, and so the pattern of thickening alone cannot be used for differential diagnosis [50]. CMR findings are often accompanied by signs of fluid overload in the form of pleural or pericardial effusions (Table 4A,C), but these may not be seen in early disease. Efforts to differentiate between ATTR and AL using CMR have been made, and comparisons show more extensive LGE, LGE of the RV, and higher LV mass in ATTR versus less extensive more subendocardial LGE in AL [51]. The overlap between AL and ATTR amyloidosis, though, remains substantial. ATTR cardiac amyloid can also be readily identified by ^99m^DPD bone scintigraphy, particularly in the absence of light chain excess or evidence of a paraprotein or plasma cell dyscrasia [52]. Emerging PET tracers, such as delayed [18F]-florbetapen cardiac uptake, represent promising biomarkers, which may discriminate cardiac amyloid infiltration due to AL from ATTR and in future may further obviate the need for endomyocardial biopsy [53].

## 13. Hypertrophic Cardiomyopathy

In clinical practice, the differentiation of hypertrophic cardiomyopathy from hypertensive LVH is a frequent and difficult challenge, especially as the two pathologies frequently coexist. Hypertrophic cardiomyopathy is typically characterised by asymmetrical LVH, usually involving the septum, but any myocardial segment can be involved [54]. Hypertensive LVH often causes concentric LV remodelling, but asymmetrical LVH is not infrequent, and concentric hypertrophic cardiomyopathy is well-described particularly in non-sarcomeric phenocopies (Table 4D), so the pattern of remodelling alone may not be helpful unless it disproportionately affects, for instance, apical segments that do not hypertrophy in isolation in patients with HHD [55,56]. Patients with sarcomeric HCM also frequently develop a reverse septal curvature morphology with a loss of concavity of the septal endocardial surface (Table 4E,F) [57]. Other features that may point towards HCM as opposed to hypertensive heart disease include ancillary abnormalities such as elongation of the anterior leaflet of the mitral valve [58]; protrusion of the anterior leaflet into the LV cavity–26 mm or more (“night cap” mitral valve) [59]; the presence of numerous myocardial crypts [Table 4G] [59] (isolated myocardial crypts are likely within normal limits [60]); apical displacement of the papillary muscles; anteromedial displacement or duplication of the anterolateral papillary muscles; apico-septal muscle bundles (Table 4H) [61]; and accessory papillary muscles or anomalous direct insertion of papillary muscles onto the mitral valve [62]. Systolic anterior motion of the anterior leaflet of the mitral valve and the associated posteriorly directed MR and left ventricular outflow tract obstruction are more frequently seen in HCM than hypertensive heart disease but can occur in both settings, particularly if there is hypertension and isolated basal septal hypertrophy with hyperdynamic LV contractility in the elderly [63]. Some degree of LGE can be seen in approximately two-thirds of patients with HCM, most often in the areas of maximum hypertrophy [64]. The pattern and extent of enhancement are often very heterogeneous within as well as between patients [65]. Diffuse patchy mid-wall enhancement is frequently seen in advanced hypertensive heart disease with poor blood pressure control (Table 4I) but equally can be seen in HCM [47]. However, very dense organised fibrosis is more commonly seen in the latter, particularly in the so-called burned-out phase (Table 4J).

## 14. Fabry’s Disease

CMR can also be used to identify several HCM spectrum disorders or phenocopies. Patients with Fabry’s disease in the early phases of cardiac involvement can display a drop in native *T*1 as glycosphingolipids accumulate in the myocardium (in contrast to normal or increased native *T*1 typically seen in sarcomeric HCM or hypertensive heart disease) [66]. However, as the disease progresses, these values can pseudonormalise and as overt fibrosis supervenes, late enhancement can develop, usually in the basal inferolateral wall (Table 4K) (an unusual location for fibrosis in sarcomeric HCM) [66]. Fabry’s disease can be diagnosed by measuring alpha galactosidase enzyme activity in male patients, but genetic testing is required to confirm the diagnosis and reliably detect carrier status in females [67]. CMR can be useful to raise the suspicion of Fabry’s and to detect cardiac involvement in hemizygous males or female carriers but should not be relied upon in isolation for diagnosis or exclusion.

## 15. Danon Disease

Other X-linked storage disorders such as Danon disease also have characteristic LGE findings (dense LV endocardial enhancement) that allow their differentiation from sarcomeric HCM (Table 4L) [68], although the latter can be readily diagnosed by clinical/neurological evaluation of the patient as well as by diagnostic genetic testing.

## 16. Cardiac Sarcoidosis

Sarcoid can occasionally cause LV thickening when there is focal or extensive granulomatous infiltration of the myocardium [69]. If there is significant myocardial oedema, this can itself give rise to pseudohypertrophy [69]. The identification of ancillary imaging findings (e.g., significant mediastinal lymphadenopathy, unexplained hepatosplenomegaly) and myocardial oedema imaging (T2W-spin echo sequences or parametric mapping), as well as clinical findings or FDG-PET-CT, can achieve a differential diagnosis [41].

## 17. Athlete’s Heart

The extent/severity of hypertrophy can be of value in a differential diagnosis. It is unusual for hypertensive heart disease to produce very severe hypertrophy (>20 mm) and never extreme hypertrophy (>30 mm). Similarly, it is often suggested that high levels of athletic activity can be associated with LVH. However, most patients who experience athletic remodelling develop mild symmetrical cavity dilatation and modest increases in LV mass. Increases in LV wall thickness are uncommon and, if present, tend to be very mild (13–15 mm). Such changes are more commonly seen in athletes with black African ancestry. Increases in LV wall thickness can often be seen in recreational bodybuilders who take androgens or an exogenous growth hormone [70]. Interestingly, recreational bodybuilders who undertake similar levels of isometric exercise but who do not use exogenous trophic substances seldom appear to demonstrate increases in wall thickness [70].

As LVH increases, there is a reduction in ECV in athletes (cellular hypertrophy) but an increase in ECV in patients with HCM (cellular disarray and extracellular matrix expansion). Based on this divergent finding, ECV may be helpful to distinguish HCM and athletic remodelling, particularly in subjects with indeterminate maximal wall thickness (13–15 mm) [71].

## 18. Calcium Score and Computed Tomography Coronary Angiography in HHD

The relationship between hypertension grade and coronary atherosclerosis was evaluated in 8238 patients who underwent calcium scoring and computed tomography coronary angiography (CTCA) for screening purposes. The prevalence of calcified and non-calcified plaques increased with the grade of hypertension and was 17%, 28%, 34%, and 40% in normotensive, pre-hypertension, stage 1 hypertension, and stage 2 hypertension, respectively (*p* < 0.05) [72].

CTCA has the highest negative predictive value of all imaging techniques regarding the exclusion of macrovascular CAD. Often coronary artery tortuosity can be documented (Table 4M). Arterial tortuosity is often associated with arterial hypertension in addition to other factors such as female sex, older age, and other cardiovascular risk factors [73]. In a study population with a high percentage of patients with hypertension (72%), the prevalence of coronary artery tortuosity was 39%; moreover, hypertension was an independent predictor of coronary artery tortuosity in these patients and was associated with increased risk of lacunar infarction [74].

## 19. Nuclear Cardiology in HHD

The correlation between blood pressure and myocardial metabolism has been evaluated in 86 individuals undergoing fluorodeoxyglucose positron emission tomography (18-F-FDG-PET) PET [75]. This isotope is easily taken up by the (hypertrophied) heart, similar to cancer metabolism where 18-F-FDG shows increased uptake in areas of cell growth and proliferation. 18-F-FDG uptake correlated with patients’ systolic, diastolic, and mean blood pressure [75].

Apart from diagnosing macrovascular disease/ischaemia in patients with HHD, myocardial perfusion reserve (MPR) assessed by Rubidium (Rb)-82 PET can be used to risk stratify patients with hypertension. In a study of 517 hypertensive patients undergoing Rb-PET imaging, the 26% of patients who had resistant hypertension had more frequent LVH, lower myocardial blood flow rates, and lower MPR than subjects without resistant hypertension. Age, resistant hypertension, and impaired MPR were independent predictors of adverse events over a median follow-up of 38 months [76].

## 20. Extracardiac Imaging for Identification of Secondary Hypertension and Sequelae of Long-Standing Hypertension

Secondary hypertension affects approximately 5–10% of the general hypertensive population [77]. Imaging may play an important role in the detection and subsequent treatment of both endocrine (such as hyperaldosteronism, phaeochromocytoma, or hyperparathyroidism) and non-endocrine secondary hypertension [78].

The most common cause of primary hyperaldosteronism is an aldosterone-producing adrenal adenoma. Adrenal adenomas usually present as round or oval masses. Using a threshold value of ≤10 Hounsfield units (HU) at non-contrast CT leads to high sensitivity and specificity in their detection [79]. Given their fatty content, adrenal adenomas may also be detected using chemical shift imaging with characteristic signal drop-outs on in- and opposed-phase imaging [80].

Phaeochromocytomas are a rare cause of secondary hypertension and account for only <5% of patients with secondary hypertension. These tumours usually present as large, heterogeneous masses with both necrosis and cystic changes and avid contrast enhancement [81].

Common non-endocrine causes are aortic coarctation (Table 4N) and renovascular hypertension caused by diseases such as fibromuscular dysplasia (FMD) or renal artery stenosis.

Aortic coarctation is defined as luminal narrowing near the origin of the left subclavian artery and ligamentum arteriosum and is a very rare cause of secondary hypertension with only 0.2% of patients with hypertension affected [82]. Increased afterload due to mechanical obstruction and potential renal ischaemia are regarded as underlying pathomechanisms. CT and, particularly for follow-up, MR angiography are the optimal imaging methods for the detection and quantification of coarctation.

Regarding renovascular hypertension, FMD or renal artery stenosis are the most common causes (Table 4O). Both conditions lead to decreased renal perfusion and subsequent increased systemic blood pressure, but have a different appearance in terms of location: While (atherosclerotic) renal artery stenosis most commonly affects the proximal renal artery, FMD affects the middle segments with alternating strictures and dilatation [83].

Patients with long-standing hypertension often also develop increased arterial stiffness with accelerated aortic pulse wave velocity as measured by CMR velocity-encoded imaging. A stiff aorta may further increase LV afterload [31].

## 21. Limits and Caveats in Imaging

All markers of cardiac damage in HHD are influenced by age, gender, and ethnicity. Normalisation to body surface area may be misleading in obese individuals; normalisation for body height, a good surrogate of fat-free mass, appears to be more acceptable but used less frequently in clinical practice [25]. All imaging findings should always be interpreted in the broader clinical context of the patient including patient history, ECG, and blood biomarkers.

## 22. Conclusions

Due to the high prevalence of hypertension, HHD is one of the most common heart diseases and a frequent cause of diastolic heart failure and LV impairment. In clinical practice, comprehensive echocardiography may be sufficient to recognize most features of HHD. In patients with LV hypertrophy disproportionate to the degree of hypertension and patients where dual pathology is assumed, tissue characterization with CMR can be very helpful in the differential diagnosis, risk stratification, and also to detect extracardiac pathology associated with HHD.

## Figures and Tables

**Table 1:** Comprehensive examination of patients with arterial hypertension for identification of syndromic causes, risk factors for secondary hypertension, and to screen for cardiovascular sequelae.

Symptoms/Review of Systems	Rationale
Renal disease	Cause/complication of hypertension
Coarctation of the aorta	Cause of secondary hypertension
Endocrinopathies -Conn’s syndrome; Cushing’s disease/syndrome; Phaeochromocytoma; Primary hyperparathyroidism; Acromegaly	Cause of hypertension
Fibromuscular dysplasia	Cause of hypertension
Vascular Risk Factors	Risk factors for complications
Obesity	Cause for hypertension and risk factor for complications
Hyperuricaemia/gout	Part of metabolic syndrome
Diabetes	Risk factor; part of the metabolic syndrome
Past history of TIA or stroke	End-organ damage
Obstetric History (for female patients)–gestational hypertension; pre-eclampsia	Risk factors for developing hypertension
Past history of migraine with aura	Vascular risk factor but may also influence choice of drug therapy
**Social History/Risk factors for developing hypertension**	
Occupation	Sedentary versus manual work; night-shift work/sleep hygiene–risk factors for hypertension and complications
Smoking history	Risk factor for complications
Alcohol	Cause of hypertension
Illicit Drug Misuse - Cocaine, amphetamines, anabolic steroids, growth hormone	Cause of hypertension
Exercise and Diet history	Physical inactivity as a risk factor and modifying dietary intake of salt or saturated fat to modify cardiovascular risk
**Family History**	
History of hypertension	May suggest familial hypertension or tendency to metabolic syndrome
History of heritable syndromes associated with hypertension, e.g., multiple endocrine neoplasia; polycystic kidney/heritable renal disease, Fabry’s disease; congenital heart disease; hypertrophic cardiomyopathy (as a cause of LVH)	To guide investigations/diagnostic testing and to inform the need for wider family screening
**Drug History**	
Sympathomimetics (e.g., phenylephrine, ephedra alkaloids); Corticosteroids; Anabolic Steroids; Growth Hormone; Liquorice; Oral contraceptive Pill; NSAIDs; Erythropoietin	Potentially addressable causes

**Table 2:** Key clinical features to assess in patients with arterial hypertension.

Inspection	Rationale
Cushingoid facies, acromegalic facies, centripetal obesity, tendon xanthomata/stigmata of hyperlipidaemia	Markers for secondary causes of hypertension and increased cardiovascular risk
Height/weight/Waist circumference	To calculate body mass index; assess centripetal obesity
**Palpation**	
Peripheral pulses	Atrial fibrillation (as a complication of chronic hypertension); to screen for peripheral vascular disease
Radio-femoral delay	Coarctation
Jugular Venous Pressure	Signs of heart failure/elevated systemic venous pressures
BP pressure measurement Ankle Brachial Pressure Index	To diagnose hypertension and to screen for peripheral vascular disease. Should be done after at least 5 min at rest in a sitting position with the arm supported and using an appropriately sized cuff and oscillotonometric equipment. On first attempt, should be done in both arms to document any difference present.
Apical rate, rhythm, position, and character (? heaving apex beat)	Indicative of a pressure loaded hypertrophied left ventricle; apical displacement may suggest LV dilatation
**Auscultation**	
Heart for heart sounds/murmurs, great vessels for bruits, chest for coarse inspiratory crackles indicative of left ventricular failure)	To screen for 4th heart sound (indicative of LVH), and 3rd heart sound (which may suggest LV dysfunction). To screen for valvular heart disease (? Ejection click and systolic murmur associated with bicuspid valve; ejection sound associated with coarctation); pansystolic murmur in the mitral area associated with functional MR.
Fundoscopy	To assess for features of hypertensive retinopathy and background changes which may suggest comorbid diabetes).

**Table 3:** The cardiovascular magnetic resonance protocol used for assessment of hypertensive patients. The described protocol can be acquired within 45–50 min. Images are examples from patients with hypertensive heart disease.

CMR Imaging Protocol for Hypertensive Heart Disease and Differential Diagnosis			
CMR Sequence	Possible Findings	Examples	
**Localisers/Scout** **Images** 3′	**Extracardiac findings** Aortic aneurysm Coarctation of the aorta Atherosclerosis of vessels Adrenal mass	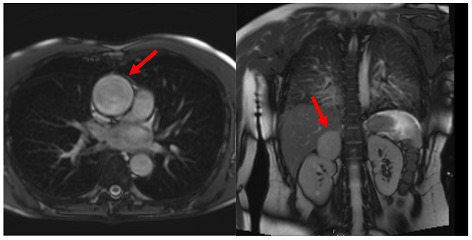	
		Aneurysm ascending aorta (52 × 52 mm)	Phaeochromocytoma
**Long axis cine** **(4Ch, 2Ch, 3Ch)** 3′	**Anatomy and Function** Systolic function LV hypertrophy LVOT obstruction Systolic anterior motion of anterior mitral leaflet/chordae Left atrial dilatation Strain analysis	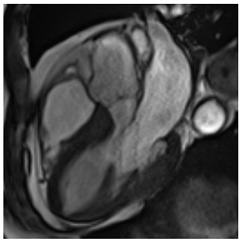	
		Concentric LV hypertrophy with sigmoid shaped basal septum	
**Native T1 maps** 5′	**Tissue characterization** Focal or generally elevated T1 values suggesting focal or diffuse fibrosis	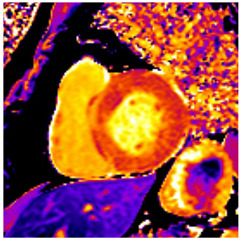	
		Mildly elevated T1 values in basal inferior/inferolateral segment	
**Vasodilator stress perfusion** 5–8′	**Ischaemia** Significant coronary artery disease (regional perfusion defect) Microvascular dysfunction (circumferential perfusion defect)	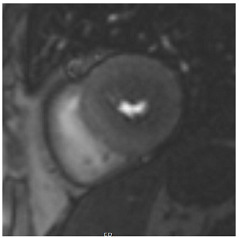	
		Microvascular dysfunction	
**Cine imaging** **(short axis stack)** 10′	**Anatomy and Function** LV function LV hypertrophy Strain analysis	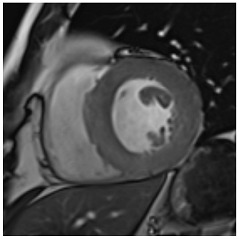	
		Concentric hypertrophy with wall thickness 13–14 mm	
**Rest perfusion** 4′	**Ischaemia and Scar** Discrimination of ischaemia from artefacts	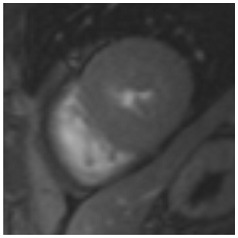	
		No artefacts detected on rest perfusion (same patient as above)	
**LGE imaging** 10′	**Tissue characterisation** Patchy, non-ischaemic fibrosis Myocardial infarction	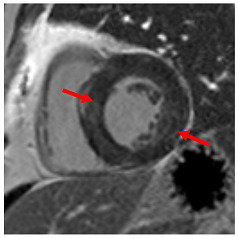	
		Subtle patchy, non-ischaemic LGE in basal septal and inferolateral segments	
**Post-contrast T1/extracellular volume (ECV) mapping** 5′	**Tissue characterisation** Borderline or elevated ECV Focal fibrosis	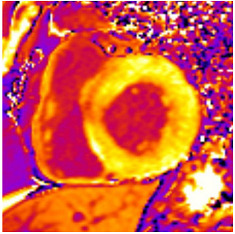	
		Borderline ECV	

**Table 4:** Example images of various differential diagnoses of hypertensive heart disease.

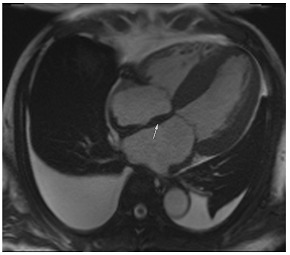	**A:** 4 chamber cine from a patient with hereditary ATTR cardiac amyloidosis. Note the marked thickening/infiltration of the atrial walls and interatrial septum (white arrow) and the diffuse left ventricular thickening. There are also significant bilateral pleural effusions indicating a degree of cardiac decompensation.	
**B:** Late gadolinium enhancement sequence (mid-ventricular short axis) from a patient with diffuse myocardial infiltration with sarcoid (proven on cardiac biopsy). There is marked septal hypertrophy and there are multiple distinct predominantly epicardial or mid-wall foci of enhancement in keeping with granulomatous infiltration.		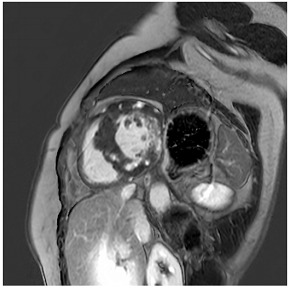
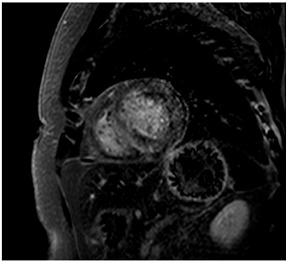	**C:** Late gadolinium enhancement sequence (basal short axis) showing abnormal gadolinium kinetics with subendocardial enhancement involving the LV sub-endocardium and the RV aspect of the septum (“zebra sign”).	
**D:** Late gadolinium enhancement sequence (4 chamber) in a patient with mitochondrial disease (a concentric hypertrophic cardiomyopathy phenocopy). There is very feint diffuse septal mid-wall enhancement on a background of concentric left ventricular hypertrophy. There is also an associated moderate pericardial effusion.		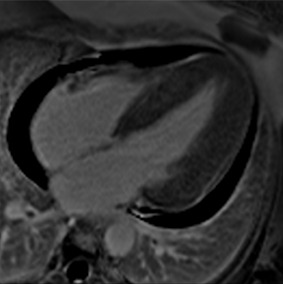
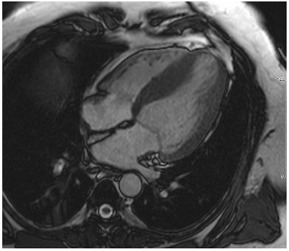	**E:** Four chamber cine from a patient with sarcomeric hypertrophic cardiomyopathy. There is loss of normal septal concavity (reverse septal curvature morphology).	
**F:** Late gadolinium enhancement sequence for patient in **E** above. There is patchy mid-wall fibrosis in the hypertrophied septum (large arrow). Hypertrophy at the mid-ventricular level has given rise to mid-cavity gradient which in turn has caused sub-endocardial injury/fibrosis of the apical segments (small arrows).		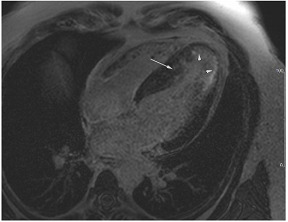
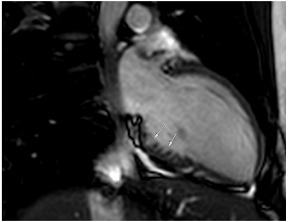	**G:** Two chamber cine from a patient with a family history of hypertrophic cardiomyopathy who is a carrier of a pathogenic sarcomere mutation. There are multiple inferior crypts (arrows) but the LV walls are of normal wall thickness or even thin. The patient is otherwise phenotype negative for hypertrophic cardiomyopathy.	
**H:** Three chamber cine sequence from a patient with sarcomeric hypertrophic cardiomyopathy. There is asymmetrical septal hypertrophy with an associated apico-septal muscle bundle (arrow).		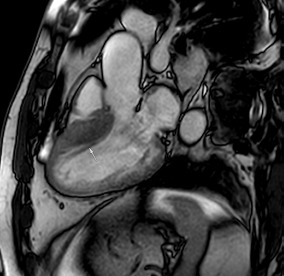
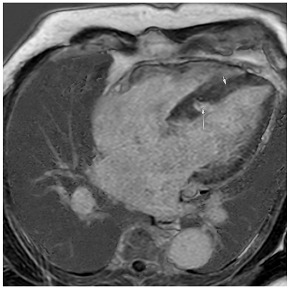	**I:** Four chamber late gadolinium enhancement sequence in a patient with hypertensive heart disease. There is septal hypertrophy and diffuse mid-wall fibrosis (small arrow). In addition, there is a small focal septal infarct (large arrow), the consequence of an embolic event due to paroxysmal atrial fibrillation, a common complication of chronic hypertension. Note also the enlarged left atrium.	
**J:** Late gadolinium enhancement sequence (mid-ventricular short axis) from a patient with “burned out” or advanced hypertrophic cardiomyopathy. The onset of even mild LV dysfunction and this phenotype can be a harbinger for increased risk of malignant ventricular arrhythmias and heightened risk of progression to advanced heart failure.		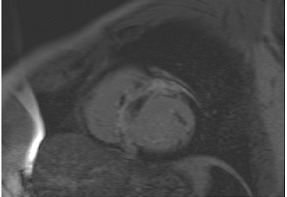
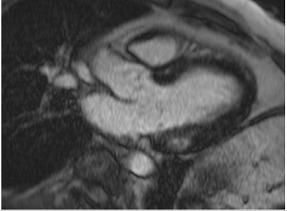	**K:** Late gadolinium enhancement sequence (3 chamber long-axis) showing focal mid-wall fibrosis in the basal inferolateral wall. This is typical of cardiac involvement in Fabry’s cardiomyopathy.	
**L:** Late gadolinium enhancement sequence (mid-ventricular short axis) showing septal hypertrophy, and dense almost circumferential subendocardial enhancement in a non-coronary pattern typical of that seen in Danon disease. There is also dense enhancement of the anterior and inferior LV/RV insertion points.		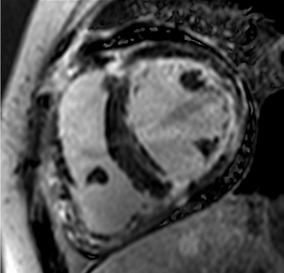
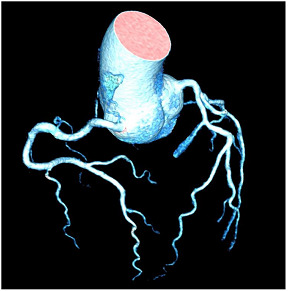	**M:** Tortuous “corkscrew” appearance of coronary arteries in a patient with hypertensvie heart diasease (3D multiplanar reconstruction of computed tomography coronary angiography)	
**N:** Patient with post-ductal coarctation of the aorta (blue arrow) and long-standing arterial hypertension		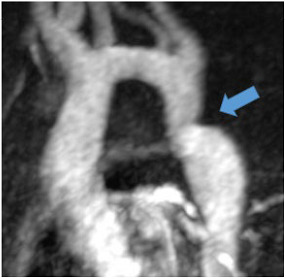
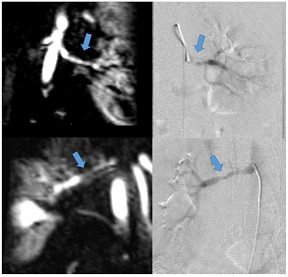	**O:** Patient with known fibromuscular dyplasia (FMD). Magnetic resonance angiography (left-sided images) and invasive angioraphy (right-sided images) of the left (upper row) and right (lower row) renal artery demonstrate typical “string-of-beads” appearance (blue arrows) of FMD.	

**Table 5:** Differential diagnosis in LV hypertrophy. Other rare causes of LV hypertrophy not considered in this table such as rare storage disorders, mitochondrial myopathy, aortic coarctation, etc.

Differential Diagnosis in LV Hypertrophy (LVH) In Most Cases, LVH May Be Attributable to Hypertension, Valve Disease (Aortic Stenosis), or Obesity.					
	Hypertensive Heart Disease	Hypertrophic CMP (1:500)	Cardiac Amyloidosis	Anderson-Fabry Disease (1:20,000–40,000)	Athlete’s Heart
	Pathological LVH				Physiological LVH
**History**	Long-lasting hypertension	Positive family history Symptomatic LVOT obstruction (syncope, dyspnoea, chest pain) Palpitations (SV/V arrhythmias)	Multiorgan disease Heart failure (Natriuretic peptides↑) Age > 50–60 years	Multi-system lysosomal storage disease X-linked (men > women) Palpitations (SV/V arrhythmias)	High level athletic activity
**ECG**	High voltage QRS	High voltage QRS “Giant negative T waves” in apical HCM	Peripheral low voltage QRS Heart block	Young age: Short PR (no delta wave) Older age: AV block Widened, high voltage QRS	Bradycardia, AV block I High voltage QRS Early repolarisation
**Echo/** **CMR**	Wall thickness <15 mm (Black <15–20 mm) LVH↓ with OMT for hypertension	Wall thickness ≥ 15 mm, ≥13 mm (in familial HCM) Diastolic dysfunction, large atria ~60%: asymmetrical septal HCM → SAM/MR (inferolateral jet); HCM vs. HOCM ~25–30%: symmetrical concentric HCM ~10%: apical HCM ~5% mid-cavity HCM → apical pouch/LGE	Longitudinal contraction↓ Diastolic dysfunction, large atria Global LV (and RV) concentric thickening Pericardial and pleural effusions	Diastolic dysfunction, large atria Concentric LVH Patterns of hypertrophy often indistinguishable from HCM.	Symmetrical chamber dilatation and LVH Mild atrial enlargement but normal diastology Max. wall thickness ≤ 12 mm (top athletes < 14–16 mm) LVH↓ after deconditioning
**CMR** **Tissue characterisation**	Diffuse LGE in non-specific pattern possible in up to 50% with HHD	Patchy LGE of RV hinge points LGE/ECV↑ in areas of maximum hypertrophy CMR ICD/SCD risk factors: (I) LVEF < 50%; (II) LGE extent > 15%; (III) apical aneurysm	ECV ≥ 40% (amyloid burden) Difficulty in nulling Global subendocardial distribution (non-coronary pattern) DPD bone scintigraphy for ATTR CA	Low native T1 (early phase lipid accumulation) Basal inferolateral LGE (late phase) Genetic testing/enzyme replacement therapy	Usually no LGE

## Data Availability

Not applicable.

## Supplementary Materials

The following supporting information can be downloaded at: https://www.mdpi.com/article/10.3390/jcm12093122/s1, Table S1: Electrocardiograms in various forms of LV hypertrophy.

## Abbreviations

CMR	Cardiovascular Magnetic Resonance
GLS	Global Longitudinal Strain
HFpEF	Heart Failure with preserved Ejection Fraction
HFrEF	Heart Failure with reduced Ejection Fraction
HHD	Hypertensive Heart Disease
LA	Left Atrium/Atrial
LAVI	Left Atrial Volume Index
LGE	Late Gadolinium Enhancement
LV	Left Ventricle/Ventricular
LVDD	Left Ventricular Diastolic Dysfunction
LVEF	Left Ventricular Ejection Fraction
LVH	Left Ventricular Hypertrophy
LVM	Left Ventricular Mass
LVMI	Left Ventricular Mass Index
TTE	Transthoracic Echocardiography
