# Supplementary material for: Hypertensive Heart Disease—The Imaging Perspective
Source: J Clin Med. 2023 Apr 25;12(9):3122. doi: 10.3390/jcm12093122 (PMC10179093; doi:10.3390/jcm12093122)
Supplement: Supplementary file 1 [file jcm-12-03122-s001.zip › jcm-2305075-supplementary.pdf]

Table S1. Electrocardiograms in various forms of LV hypertrophy.

**Hypertensive Heart Disease**<br>• High voltage QRS	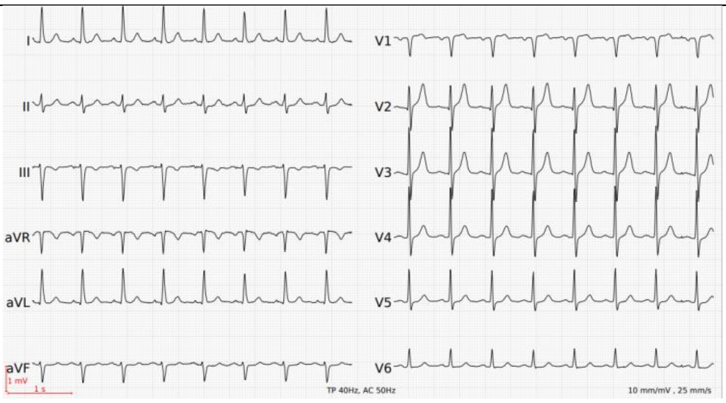
**Apical Hypertrophic Cardiomyopathy**<br>• "Giant negative T waves"<br>• High voltage QRS	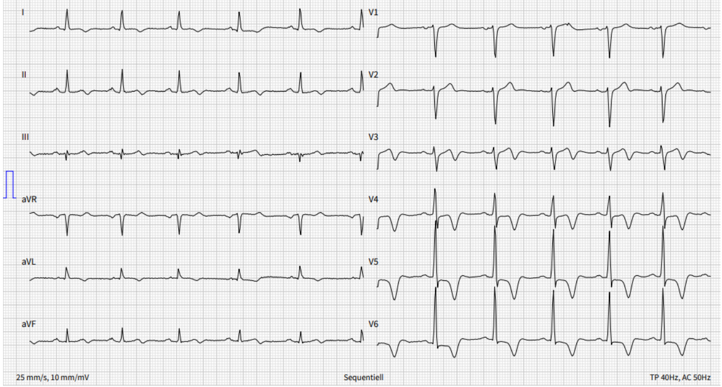
**ATTR Cardiac Amyloidosis**<br>• Peripheral low voltage<br>• Pseudoinfarct pattern in the anteroseptal leads	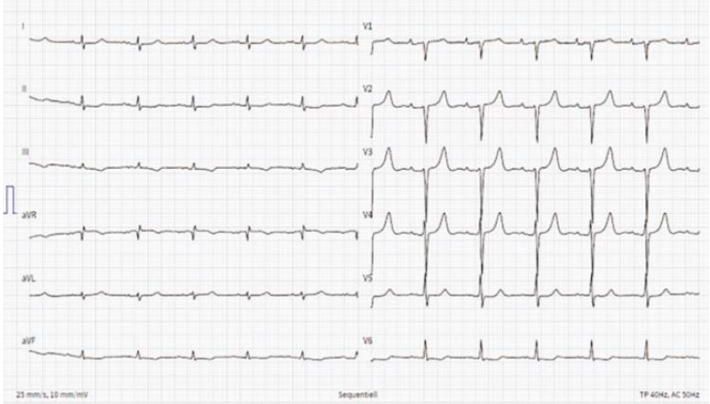
**Anderson-Fabry disease**<br>• High voltage QRS<br>• Deep T wave inversion<br>• Sinus node dysfunction requiring atrial pacing	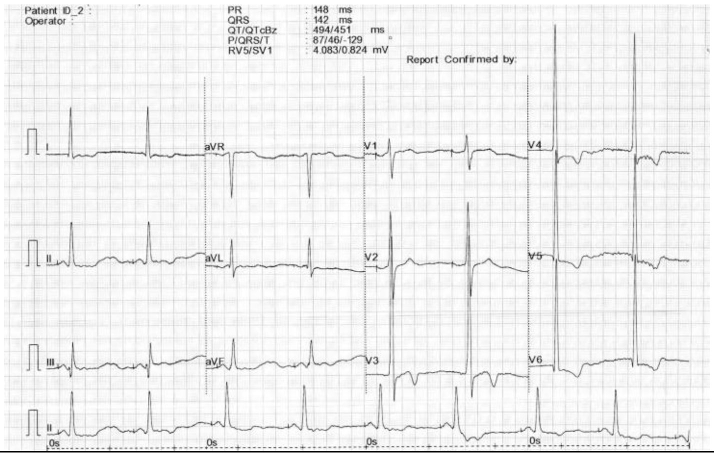

**Athlete's heart**<br>• High voltage QRS<br>• Repolarisation disorder	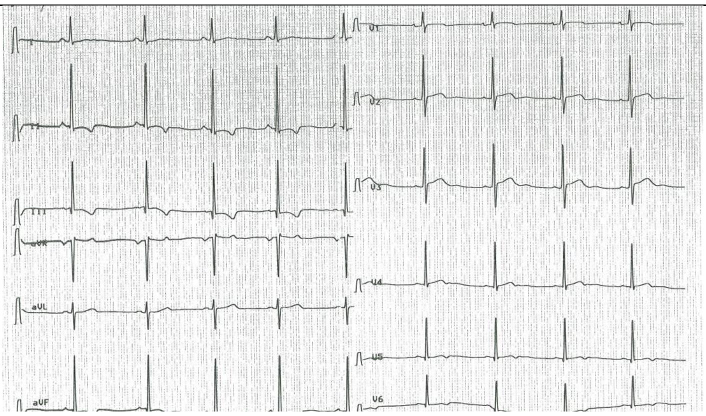
